# Integration of Postbiotics in Food Products through Attenuated Probiotics: A Case Study with Lactic Acid Bacteria in Bread

**DOI:** 10.3390/foods13132042

**Published:** 2024-06-27

**Authors:** Javier Morán, Alina Kilasoniya

**Affiliations:** 1 Department of Food Innovation, Universidad Católica San Antonio de Murcia, 30107 Murcia, Spain; 2 International PhD School, Universidad Católica San Antonio de Murcia, 30107 Murcia, Spain

**Keywords:** postbiotics, lactic acid bacteria (LAB), sourdough, bread production, food safety, European Union regulations, microbial quantification, Next-Generation Sequencing

## Abstract

The study examines the integration of postbiotics in food products through the use of attenuated probiotics, specifically lactic acid bacteria (LAB) in bread. Postbiotics, non-viable microorganisms or their metabolites, offer health benefits similar to probiotics without the risks associated with live bacteria. This research evaluates the regulatory aspects and safety of LAB in sourdough bread production, highlighting their historical and significant use in Europe before 1997. The study includes microbial quantification and Next-Generation Sequencing (NGS) to identify LAB in traditional sourdough, comparing them with historical and current EFSA Qualified Presumption of Safety (QPS) lists. Findings show that the LAB present in sourdough have been extensively and safely used in bread making, supporting their classification as non-novel foods under EU regulations. The stability and consistency of LAB metabolites in sourdough bread are also confirmed, ensuring quality and safety in each batch. The study concludes that LAB in sourdough, when inactivated through bread-making processes, are not considered novel foods, aligning with historical, scientific, and regulatory evidence.

## 1. Introduction

The growing concern for health and food safety has driven research into alternatives to traditional probiotics. Postbiotics, defined as non-viable microorganisms or their metabolites that offer health benefits, have emerged as a promising solution. Unlike probiotics, postbiotics do not present the risks associated with the viability of live bacteria, making them particularly attractive for use in foods. In the food industry, postbiotics can enhance product functionality without compromising consumer safety.

This study focuses on the integration of postbiotics in food products using lactic acid bacteria (LAB) in bread. By evaluating regulatory and safety aspects, this research not only expands knowledge on the application of postbiotics in baking but also provides a solid scientific basis for their future incorporation in other food products. Additionally, this article defines a new pathway for manufacturing postbiotics that are not considered novel foods under current regulations, thereby addressing significant concerns regarding their regulatory status. Until now, there have been uncertainties about how to classify and regulate these compounds, and this study offers a viable and scientifically justified solution to overcome these regulatory challenges.

Thus, this work not only addresses a current scientific need but also has the potential to influence the food industry and regulatory policies, offering an innovative and practical perspective for the use of postbiotics in foods.

To ensure a comprehensive and relevant selection of literature for this article, specific search terms were utilized in PubMed. The search strategy included the following terms: (Sourdough[Title]) AND (Lactic Acid Bacteria[Title]); (Sourdough[Title]) AND (Lactic Acid Bacteria[Title/Abstract]); (Sourdough[Title/Abstract]) AND (Lactic Acid Bacteria[Title/Abstract]); ((sourdough[Title]) AND (Lactic Acid Bacteria[Title])) AND (Bread[Title]); ((sourdough[Title/Abstract]) AND (Postbiotic[Title/Abstract])) AND (Bread[Title/Abstract]); (Postbiotic[Title]); and (Postbiotic[Title]) AND (Probiotic[Title]). 

These search terms were chosen to capture a wide range of studies focusing on sourdough and LAB, as well as postbiotics and their relationship with probiotics. The goal was to include studies that provide insights into the integration of postbiotics in food products, particularly those involving lactic acid bacteria in sourdough bread. By using these specific search terms, we ensured that the literature review was thorough and included the most relevant and up-to-date scientific research available.

## 2. Attenuated Microorganisms

The definition of probiotics as “live microorganisms that, in adequate amounts, confer a health benefit” emphasizes the need for viability. However, there is evidence that inactivated microorganisms can also be beneficial for health, as some effects do not depend on viability [1,2,3]. Paraprobiotics, or “inactivated probiotics”, are non-viable microorganisms that, when administered in sufficient quantities, offer health benefits. These may be safer than live probiotics, especially for individuals with compromised immune systems [4,5,6]. Postbiotics are bioactive compounds produced by probiotic bacteria that have beneficial effects even when the bacteria are not alive. These include short-chain fatty acids, enzymes, and peptides, among others, and can regulate health and maintain a healthy microbiome without the risks associated with live bacteria [7,8,9]. Therefore, postbiotics and paraprobiotics represent an opportunity to develop safe and effective functional foods, avoiding the risks of live probiotics.

The integration of postbiotics into food products, especially through the use of lactic acid bacteria (LAB) in baking, has been the subject of several scientific studies. These studies have explored various aspects, from health benefits to mechanisms of action and regulatory implications. Below is a table summarizing the main scientific studies on this topic. This table provides a deeper and updated context, highlighting key findings that support the application of postbiotics in the food industry (Table 1).

## 3. Regulatory Aspects of Attenuated Microorganisms

In the past fifteen years, various definitions of probiotics have been proposed, some of which even include non-viable microbial cells. For instance, in 1997, Reuter described probiotics as “a microbial preparation containing live and/or dead cells, including their metabolites, aimed at improving the microbial or enzymatic balance on mucosal surfaces or stimulating immune mechanisms” [12]. Similarly, in 1999, Salminen defined probiotics as “preparations of microbial cells or components of microbial cells that have a beneficial effect on the health and well-being of the host” [13]. Despite the potential legitimacy of these definitions, the current definition of probiotics, at least in Western countries, is almost unanimously accepted by the FAO/WHO as “live microorganisms which, when administered in adequate amounts, confer a health benefit on the host” [14]. Consequently, national/governmental institutions are introducing the FAO/WHO definition into their guidelines for probiotics. For example, this definition is proposed in the guidelines of the Italian Ministry of Health, in a dossier by the French Food Safety Agency, and in a guidance document supported by the Dutch Agency [15,16,17].

Probiotics available on the market should be considered as a first-generation means to correct microecological disorders, but this is not always sufficient. This has led many researchers to develop inactivated microorganisms or, further still, to select bioactive metabolic compounds present in the bacteria, which are signaling molecules with a specific chemical structure that can optimize the physiological functions of the host, regulatory reactions, metabolic, and/or behavioral actions related to the activity of the host’s indigenous microbiota. This has some advantages due to their exact chemical structure, well-dosed, very safe, and long shelf-life. This is no longer considered a myth but the result of the natural evolution of the probiotic concept [18,19,20,21].

Therefore, if the use of the word “probiotic” should be restricted to products containing live microorganisms, we will need a new terminology to unambiguously define the use of non-viable microorganisms or microbial fractions that can positively affect health. For this purpose, the terms “paraprobiotic” and “postbiotic” have been proposed to define, respectively, non-viable microbial cells (intact or broken) or crude cell extracts (i.e., with complex chemical composition), which, when administered orally in adequate amounts, confer a benefit to the human consumer. Moreover, once a health benefit is demonstrated, the assignment of a product to these categories should not be influenced by the methods used for microbial cell inactivation or obtaining probiotic extracts, which can be achieved through physical or chemical strategies, including heat treatment, γ or UV rays, deactivation, chemical or mechanical alteration, pressure, lyophilization, or acid deactivation [22,23].

Traditionally, probiotics based on live microorganisms are considered both beneficial and safe. Unfortunately, some microorganisms with known health benefits can cause opportunistic infections, increase the incidence of allergic sensitization and autoimmune disorders, produce microecological imbalance, modify gene expression, transfer antibiotic-resistant and virulent genes, cause disturbances in the integrity of the epigenome and genome, induce damage to chromosomal DNA, and activate signaling pathways associated with cancer and other chronic diseases [24].

Overall, postbiotics possess various attractive properties such as clear chemical structures, safe dosage parameters, and longer shelf life (up to 5 years when used as ingredients for foods and beverages or as nutritional supplements), which are highly sought after. Moreover, research conducted by Shenderov revealed that postbiotics have favorable absorption, metabolism, distribution, and excretion capabilities, indicating a high capacity to send signals to various host organs and tissues, triggering several biological responses. Another significant advantage of postbiotics is their favorable safety profile, as there is no need for the absorption of billions of live microbes. Furthermore, postbiotics can be applied in a controlled and standardized manner, whereas, in the case of live bacteria application, the level of active structure in the intestine depends on the respective strain’s number and metabolic activity. Therefore, specific soluble factors from particular bacteria can become a class of bacterial biological strategy for treating many diseases [25].

Additionally, the definition of postbiotic was recently defined by a panel of scientists convened by the International Scientific Association for Probiotics and Prebiotics (ISAPP) as “a preparation of inanimate microorganisms and/or their components that confers a health benefit on the host”. This definition focused on the progenitor microbial cell or cell fragments, not just the metabolites, proteins, or carbohydrates they might produce. Although such constituents produced by microbes can be functional ingredients in the preparation, they do not need to be present in a postbiotic according to this definition. In this context, previously used terms like paraprobiotics, ghostbiotics, thermoinactivated probiotics, non-viable probiotics, cell fragments, or cell lysates, among others, align with the term postbiotics as conceived by this definition [11].

Evidence is accumulating that inanimate microorganisms and/or their components can confer health benefits when administered in adequate amounts to a host. There is also research supporting the concept that microbial-derived metabolites administered to a host can elicit some beneficial physiological effects. Postbiotics bring together these two aspects of microbial influence on health into products that can be developed as foods, therapies, and other types of products to be administered for health outcomes. Mechanistic research points to significant small molecules derived from microbes, such as neurochemicals, short-chain fatty acids, defensins, bacteriocins, and others produced in situ, which are likely to mediate the many functions exerted by colonizing microbes on physiological function. However, this microbial activity is distinct from postbiotics, which must be administered to a host. Various terms have been used over the years to address this evolving research area. To facilitate communication to health professionals, industry, regulators, and the general public, coming together under a well-defined consensus definition will clarify what fits and what does not fit within the postbiotic category. It will also enable better tracking of scientific articles for future systematic reviews and meta-analyses on the topic [26].

At the regulatory level, three inanimate bacteria have been approved as novel foods (*Bacteroides xylanisolvens*, *Akkermansia muciniphila*, and *Mycobacterium setense manresensis*) [27,28,29]. The production of *B. xylanisolvens DSM 23964* includes fermentation with sugars, recovery and concentration of the culture by ultracentrifugation, and use as a starter culture for milk fermentation or storage by freezing and lyophilization. The culture’s identity is confirmed by PCR. The process follows quality standards (GMP and HACCP) and is considered safe by the EFSA, although *B. xylanisolvens* has no history of food use in the EU and was not included in the Qualified Presumption of Safety (QPS) list.

*A. muciniphila MucT* degrades intestinal mucin and is produced under anaerobic conditions, followed by pasteurization and lyophilization to obtain a powder with bacterial cells. The production process includes mixing with cryoprotectants and optional stabilizers, storage under controlled conditions, and a shelf life of 1 year. The EFSA considers that the production process is sufficiently described, and that the species has been detected in human milk.

*M. setense manresensis*, isolated from the Cardener River in Spain, is used in capsules as a food supplement. The production follows HACCP certification, with bacterial culture, heat inactivation, centrifugation, and lyophilization. The final formulation is mixed with mannitol and encapsulated. Although it has no history of consumption in the EU, the EFSA considers its proposed use safe with specific consumption restrictions (no more than 14 consecutive days and a minimum interval of 6 months between consumption periods).

In summary, EFSA’s evaluations of these inactivated bacteria conclude that the described production processes are safe and well-controlled, allowing their use as novel foods under specific conditions.

Two more inquiries have been submitted within the “Novel Foods Consultation Process” [30] (Information in compliance with Article 7 of Commission Implementing Regulation (EU) 2018/456 [31]: Biomass of heat-killed *Propionibacterium freudenreichii* with increased cobalamin content and “IBalance”, a combination of 13 heat-treated microorganisms [32,33].

The biomass of heat-killed *P. freudenreichii* with increased cobalamin content is produced by fermentation in bioreactors in the presence of cobalt salts, followed by centrifugation, washing, heat-fixing, and drying. *P. freudenreichii* biomass has been consumed in the EU as part of fermented cheeses. The higher concentration of cobalamin in the biomass is considered safe, as vitamin B12 has no clearly defined adverse effects, concluding that there is no significant change in food composition.

IBalance, a combination of 13 heat-treated microorganisms, consists of lysates of bacteria and yeasts treated thermally, mixed in specific proportions, and intended as a food ingredient. The evaluation concluded that there is no history of consumption before 15 May 1997; thus it is considered a novel food under Regulation (EU) 2015/2283 [34].

Additionally, there are two ongoing evaluations [35,36].

Whole cell heat-killed non-GMM *Mycolicibacterium aurum Aogashima DSM33539* is a non-pathogenic bacterium, heat-inactivated and presented as a dietary supplement. Safety evaluation showed no adverse effects and that the product is safe for consumption, although its final evaluation by the EFSA is still in progress.

*Anaerobutyricum soehngenii CH106* is a propionate and butyrate-producing anaerobe with positive effects on intestinal and general health. Toxicity and genotoxicity studies showed no adverse effects, supporting its use as a safe food ingredient, although its evaluation is pending final acceptance by the EFSA.

These approvals reflect a shift in the perception and regulation of microorganisms in food, allowing the inclusion of inactivated bacterial cells and their bioactive components as safe and effective ingredients. This advancement not only broadens the options for developing functional foods but also enhances the safety and stability of probiotic and paraprobiotic products available to consumers.

The new Novel Foods Regulation (Regulation (EU) 2015/2283) [34] applies from 1 January 2018. The term “novel food” refers to any food that has not been used for human consumption to a significant degree within the European Union before 15 May 1997. Probiotics belong to the categories of (a) foods consisting of, isolated from, or produced from microorganisms, fungi, or algae; and (b) foods consisting of, isolated from, or produced from cell cultures or tissue cultures derived from animals, plants, microorganisms, fungi, or algae. The new centralized procedure results in a direct evaluation by the EFSA, followed by a Comitology debate and approval or denial of authorization.

As reviewed earlier, EU regulators require specific information to ensure that inactivated probiotic derivatives are safe:

1. EFSA did not consider presence in the human microbiota to demonstrate the safety of the microorganisms (for example, *B. xylanisolvens* represents approximately a quarter of all anaerobic microorganisms inhabiting the human colon and are mostly non-pathogenic commensals, and *A. muciniphila* has been detected in human milk by quantitative PCR as reported by the applicant).
2. EFSA and European Regulators considered the QPS list as the gold standard for ensuring the safe use of microorganisms (*B. xylanisolvens* is not on the QPS list and had not been evaluated under the QPS qualified presumption of safety system at the time this novel food was evaluated by Member States), and although the QPS evaluation does not take into account specific dossier data such as the production process or unpublished data, the published studies on *B. xylanisolvens* are insufficient for inclusion in the QPS list. In the case of IBalance, it was highlighted that within the combination of 13 heat-treated dead microorganisms, *Bacillus* sp. on the QPS list only applies to the production process and not as a food ingredient. The same applied to Whole cell heat-killed non-GMM *M. aurum Aogashima DSM33539* and *A. soehngenii CH106*).
3. EFSA and European Regulators also considered the production process to ensure the safety of the microorganisms (*B. xylanisolvens* uses standard dairy industry techniques. The production process of *A. muciniphila* involves anaerobic fermentation followed by pasteurization and concentration of bacterial cells. *M. setense manresensis* is a capsule composed of heat-killed, lyophilized bacteria with mannitol as a carrier agent, and the inactivation of growth and bacterial death is achieved by heating the bacterial culture to 80 °C for 32 min. The biomass of heat-killed *P. freudenreichii* with increased cobalamin content is produced by fermentation in bioreactors with cobalt salts, followed by centrifugation, washing, heat-fixing, and drying. In IBalance, the combination of 13 heat-treated dead microorganisms consists of a mixture of lysates of probiotic bacteria and yeasts, purchased from specific suppliers or individually cultured in fermenters, with each strain processed individually and then the lysates mixed).
4. EFSA and European Regulators also considered the history of safe use of the microorganism (in the case of *B. xylanisolvens*, neither the DSM 23964 strain nor any other strain of Bacteroides has a history of use in food production and consumption, and the use of Bacteroides in food production had not been reported in the EU before May 1997. *M. setense manresensis* has no history of consumption in the EU. The biomass of heat-killed *P. freudenreichii* with increased cobalamin content is not a novel food because significant consumption in the EU was established before 15 May 1997. In IBalance, a combination of 13 heat-treated dead microorganisms, some microorganisms have been used in baked and dairy products but their use as an ingredient before 15 May 1997, in the EU had not been established. Whole cell heat-killed non-GMM *M. aurum Aogashima DSM33539* and *A. soehngenii CH106* have no history of use in the EU).

In summary, the regulation of novel foods in the EU ensures the safety of inactivated probiotic derivatives by evaluating aspects such as presence in the microbiota, the QPS list, the production process, and the history of safe use.

It is clear that most probiotic products contain inanimate microorganisms, but this does not make them ‘postbiotics’. Probiotic products are correctly considered probiotics as long as they are capable of delivering the necessary dose of live cells to confer the expected health benefit. The inanimate cells in probiotic products may contribute to the health benefit provided, but few have been studied, and their contribution is not yet clear. Future research needs to understand what inanimate microbes, with or without associated metabolites, are capable of conferring a health benefit, what mechanisms are driving the benefits, and what role inanimate microbes in probiotic products can play in driving health benefits.

Manufacturers are required to market products that are safe. This includes ensuring safe levels of use, dosages, absence of toxic contaminants, toxic elements, heavy metals, and/or pesticides in finished products and their ingredients. In the case of postbiotics, the starting microorganism must be considered, ensuring the absence of toxins or virulence factors; it must be free of antibiotic activity or incapable of producing antibiotic substances; demonstrate the absence of antimicrobial resistance (especially acquired resistance); and have clear documentation of reduced toxigenicity and/or pathogenicity of the strain [37].

## 4. Proposal of a “Regulatory Case”

Following our previous publication [38] and as an educational example, we propose a regulatory challenge that aims to demonstrate that attenuated probiotics, through the same manufacturing method as sourdough bread fermented with different lactic acid bacteria (LAB), are not considered novel food.

In 2010, the European bread market was 32 million tons across the 27 EU countries. The market share between industrial and artisanal bakers was approximately 50/50, although it varied significantly between countries. For example, in Germany, the industrial sector represented 40%, in France 35%, in the Netherlands 81%, and in Spain 19%. Bread production is stable in most countries, although some show a 1–2% annual decline. The average consumption in most countries is 50 kg of bread per person per year, with Germans and Austrians being the highest consumers at 80 kg, while in Ireland it is less than 50 kg. There is a growing demand for sourdough breads, and continuous growth is expected in these products, offering opportunities for innovation. In-store bakeries continue to be a growing sector [39].

Bread is one of the most important staple foods consumed worldwide. Its traditional recipe includes cereal flour, water, salt, and a leavening agent. Lactic acid fermentation enhances the sensory properties of bread, such as its flavor and texture. In recent years, the use of sourdough has become increasingly standardized and studied, allowing interaction with microbial cultures to improve the stability and flavor of the final product. Additionally, sourdough fermentation with LAB acts as a source of proteolytic enzymes that can eliminate gluten toxicity and reduce antinutritional factors, increasing the bioavailability of minerals [40,41].

Sourdough is a complex ecosystem in which heterofermentative LAB dominate, coexisting synergistically with yeasts adapted to the predominant acidic environment. LAB in sourdough produce lactic and acetic acids, which affect the bread’s flavor and contribute to the accumulation of amino acids and peptides that enhance umami and kokumi flavors. During fermentation, LAB and yeasts produce antimicrobials, flavor compounds, and exopolysaccharides. Additionally, LAB acidification activates enzymes that release phenolic compounds and fatty acids, enhancing flavor and nutrient bioavailability [10].

In our opinion, sourdough bread cannot be considered a “novel food”. To reach this conclusion, we have considered, first, the regulatory opinions of the European Union summarized in the following table (Table 2).

Our opinion is that bread made with sourdough fermented with LAB should not be considered a novel food because its safety and historical use are well-documented: LAB are common microorganisms in food fermentation and have an extensive and safe history of use in bread production; fermentation with LAB improves the digestibility and nutritional value of bread without introducing new or unknown health risks; its traditional production process (the sourdough bread production process follows well-documented and widely used traditional techniques) does not introduce significant changes in the final composition of the product that could be classified as new under Regulation (EU) 2015/2283; LAB are inactivated during baking due to high temperatures, leaving only their metabolites, which do not pose health risks; the presence of LAB on the QPS list (the LAB present in sourdough are not considered dangerous, and many are on the EFSA’s Qualified Presumption of Safety (QPS) list, ensuring their safe use). No artificial nanomaterials are introduced in the sourdough bread production process.

Second, to justify that LAB from sourdough are not novel foods according to Regulation (EU) 2015/2283, we aim to demonstrate that they have been significantly consumed in the EU before 15 May 1997. 

Sourdough has been used since ancient times for bread making. Historical records indicate its use since at least the Egyptian era, over 4000 years ago. In Europe, the use of sourdough is a tradition dating back several centuries and was the main method of baking before the introduction of commercial yeasts in the 19th century. Despite the introduction of commercial yeast, many artisanal bakers in Europe continued to use sourdough due to its benefits in terms of flavor, texture, and bread preservation. During the 20th century, the use of sourdough was not as prevalent in industrial production but was maintained in artisanal and home baking.

In countries like France, Germany, Italy, and the Nordic countries, sourdough has been particularly popular. For example, rye bread fermented with sourdough is a traditional food in Germany (known as “Sauerteigbrot”). In France, the tradition of “pain au levain” (sourdough bread) is very strong and has been an integral part of culinary culture for centuries.

To ensure the history of consumption, we consulted with operators and federations of food business operators to verify the use of sourdough before 1997. These consultations included professional baker associations, artisanal bakeries, companies supplying baking ingredients, as well as historical studies and culinary publications. The information gathered supports the conclusion that sourdough was used significantly in the European Union before 1997.

To this end, we have conducted a review of the scientific literature prior to that date to show which LAB have been historically used in sourdough for bread production, thus ensuring their exclusion from the novel food definition (Table 3).

According to the scientific literature consulted, before 1997, the presence of the following LAB in sourdough used for bread production is documented: *Lactobacillus acidophilus*, *Lactobacillus alimentarius*, *Lactobacillus brevis*, *Lactobacillus buchneri*, *Lactobacillus casei*, *Lactobacillus cellobiosus*, *Lactobacillus curvatus*, *Lactobacillus delbrueckii* subsp. *delbrueckii*, *Lactobacillus farciminis*, *Lactobacillus fermentum*, *Lactobacillus fructivorans*, *Lactobacillus hilgardii*, *Lactobacillus homo-hoichii*, *Lactobacillus plantarum*, *Lactobacillus rhamnosus*, *Lactobacillus sakei*, *Lactobacillus sanfranciscensis*, *Leuconostoc gelidum*, *Leuconostoc mesenteroides* subsp. *dextranicum*, *Leuconostoc mesenteroides* subsp. *mesenteroides*, *Pediococcus pentosaceus*, *Pediococcus* spp., and *Weissella viridescens*.

From the analysis of scientific literature published before 1997, we can conclude that sourdough baking is an ancient traditional practice and that European communities have historically used these microorganisms routinely for dough fermentation, demonstrating significant consumption before 1997. Numerous studies and historical records document the use of certain microorganisms in bread making, and specific microorganisms have been safely and continuously consumed in traditional bakery products, supporting their exclusion from the novel food scope.

Due to their extensive and documented use in sourdough fermentation for bread production before 15 May 1997, the aforementioned microorganisms are not considered novel foods under Regulation (EU) 2015/2283. Their history of safe and significant use in the EU supports this classification.

To present a real case, we proceeded to analyze a commonly used artisanal sourdough.

To evidence the viability of bacteria in the sourdough, bacterial quantification tests were conducted. To begin, 0.1 g of sourdough sample were weighed under sterile conditions and then homogenized in sterile 1X PBS. Then, serial dilutions were performed and the samples were plated on MRS Broth with Agar Agar. The plates were incubated at 28 °C for 72 h, and colonies were counted. The results showed 5 × 10^7^ CFU/g in the sourdough, and 0 CFU/g in the baked bread, confirming the inactivation of LAB during baking.

Following this initial analysis, we proceeded with bacterial identification of complex samples by NGS sequencing (Oxford Nanopore) of artisanal sourdough. The reads obtained per sample, after filtering by size and quality, were 13,080 for sourdough, 14,575 for dough, and 14,774 for bread. The average quality of the reads is approximately 18. After obtaining the number of reads assigned to the species identified in each sample, we compared the LAB from the sourdough with those described in the scientific literature before 1997 and with the latest EFSA QPS review (Table 4).

We can confirm that all LAB from the analyzed sourdough have been described as components of sourdough used in the European Union before 1997 and/or appear in the latest revision of the QPS list, which ensures their significant consumption before 1997 in addition to their safety of use.

Finally, we would like to address a topic that might be a cause for concern regarding whether the consortia or communities of LAB in sourdough are stable over time and during the bread-making process.

Sourdough is an intermediate product for bread preparation that contains metabolically active microorganisms, being a rich source of various species and strains of LAB and yeasts, influenced by regional and artisanal factors. The LAB present can originate from natural contaminants in the flour or from starter cultures, reaching cell densities exceeding 10^8^ CFU/g. The microbial ecology of fermentation depends on endogenous factors, such as the composition of the dough, and exogenous factors, such as temperature and redox potential. Process parameters, including dough yield (water activity), salt addition, starter amount and composition, the number of propagation steps, and fermentation time, significantly influence the selection of LAB microflora and prevent the growth of other microorganisms from raw material contamination or the bakery environment [59].

Studies have shown that microbial associations in sourdough can last for years due to the selective pressure of environmental conditions. The biodiversity of LAB in sourdough can be restricted or diverse, with species from the genus Lactobacillus being the most frequent. The distribution of LAB taxa varies according to the sourdough ecosystem, influenced by ecological factors such as temperature, pH, redox potential, ionic strength, dough yield, and microbial products such as lactate, acetate, carbon dioxide, and ethanol, as well as factors resulting from the substrates present in the cereal fraction and endogenous factors and microbial enzymatic reactions [60,61].

It is important to note that many studies are based on single isolations and do not always use modern taxonomic identification techniques and polyphasic approaches, as is often the case today and in our study. Additionally, few data have been reported on the influence of time on the composition of sourdough. Finally, it should be emphasized that LAB isolated from many sourdoughs are difficult to cultivate on common laboratory media. This may be due to these bacteria being selected during repeated sourdough propagation, resulting in a flora with specialized nutrient and growth condition requirements [62,63].

From all the above, we can conclude that if the sourdough and bread-making processes are always the same, we consider that the concentration of LAB metabolites in the bread will remain constant and consistent in each batch. There are several reasons to support this idea: the manufacturing process is rigorously followed and closely monitored, so factors affecting the production of LAB metabolites, such as temperature, humidity, fermentation time, and ingredients used, will remain stable, leading to consistent LAB metabolite production in each batch. The ingredients used in bread making remain constant, contributing to uniformity in the chemical and microbiological composition of the bread in each batch. Ingredients like flour, water, and yeast can influence the metabolic activity of lactic acid bacteria, and maintaining their quality constant will help maintain consistency in metabolite concentration. The sourdough culture used for fermentation can act as a constant source of lactic acid bacteria and yeasts, favoring stability in metabolite production during fermentation. Through regular monitoring and quality control of the manufacturing process, deviations or inconsistencies in bread production between batches can be identified, allowing adjustments or corrections to maintain the desired consistency in LAB metabolite concentration [64,65].

Although this assumption is reasonable, we should conduct periodic analyses and tests to verify whether the concentration of LAB metabolites actually remains constant between different batches of bread, ensuring the quality and safety of the final product. In any case, at a minimum, we should periodically check the sensory characteristics of the bread through a trained panel that analyzes the acceptance of the sour taste, volume, chewability, swallowability, hardness, and moisture loss. These tests help us assess possible differences in carbohydrate fermentation patterns since the microorganisms impart their distinctive characteristics to the sourdough bread, with homofermentative LAB contributing to better dough softening.

## 5. Conclusions

According to Regulation (EU) 2015/2283, a novel food is any food that has not been used for human consumption to a significant degree within the European Union before 15 May 1997. Novel foods include products containing microorganisms that do not have a history of safe consumption before this date.

Sourdough is a natural fermentation technique used in baking for thousands of years. Its use in the European Union before 15 May 1997, is well-documented and recognized in European culinary tradition. Therefore, sourdough bread should not be considered a novel food under Regulation (EU) 2015/2283. Its safety and significant use are supported by scientific studies, historical records, and testimonials from experts and industry associations.

The key question we want to answer is whether the attenuated (non-viable) LAB isolated as components in traditional sourdoughs and listed in the QPS list when inactivated by a method similar to bread-making are considered novel foods under Regulation (EU) 2015/2283. In our opinion, they are not novel foods for the following reasons:

1. Traditional Production Process of Sourdough Bread: The process is traditional and well-documented. It includes fermenting a mixture of flour and water with LAB and yeasts, followed by kneading, resting, dividing, shaping, proofing, and baking. During baking, LAB are inactivated due to high temperatures and dehydration. Factors affecting LAB viability include temperature, moisture content, and bread matrix structure. However, metabolites produced by LAB during fermentation remain in the bread, contributing to its sensory and nutritional properties.
2. History of Significant Use: LAB have been traditionally used in sourdough fermentation in bread production in Europe and have a historical and documented use in the EU before May 15, 1997. This includes the presence of LAB in the EFSA’s Qualified Presumption of Safety (QPS) list. Therefore, the use of LAB in sourdough does not introduce new or unknown risks to human health.
3. Safety: LAB used in sourdough have a history of safe use in food production. Many of these bacteria are on the EFSA’s QPS list, ensuring their safety. The presence of LAB in the human microbiota and their historical use in fermented foods like sourdough bread support their safety and significant consumption.
4. Consistency of Ingredients and Processes: The use of consistent ingredients and processes ensures stability in LAB metabolite production across different batches of bread. Sourdough is fermented using well-documented traditional techniques widely employed in artisanal and home baking. During the bread baking process, LAB are inactivated due to high temperatures. Metabolites produced by LAB during fermentation remain in the bread, but the non-viable bacteria do not pose health risks.
5. Scientific Research: Historical studies and current microbiological analyses confirm the presence and safe use of LAB in sourdough. These studies have shown that LAB fermentation improves the digestibility and nutritional value of bread.
6. EFSA and other European regulators have evaluated various inanimate microorganisms as novel foods, considering aspects such as safety (QPS list), production and inactivation processes, and history of use. LAB used in sourdough have a history of safe and significant use in the EU before 1997 and are on the QPS list.
7. There is no information indicating that sourdough is used as a medicinal product according to Directive 2001/83/EC [66] in the European Union. Sourdough is exclusively used in baking to produce bread and other fermented products, and it is not promoted or administered for therapeutic or medical purposes.

Therefore, in our opinion, LAB present in sourdough when inactivated by the same bread-making process would not be classified as novel foods. Historical, scientific, and regulatory evidence supports this conclusion, ensuring that both these microorganisms and the bread produced with sourdough containing them continue to comply with current food safety regulations in the European Union.

## Figures and Tables

**Table 1:** Relevant scientific studies on the integration of postbiotics in food products.

Author(s)	Year	Type of Study	Key Findings
Kataria, J., et al. [8]	2009	Review	Postbiotics can maintain a healthy microbiome without the risks of live bacteria.
Adams, C.A. [1]	2010	Review	Postbiotics offer health benefits without the risks associated with live bacteria.
Taverniti, V.; Guglielmetti, S. [4]	2011	In vitro Study	Paraprobiotics may be safer for individuals with compromised immune systems.
Hill, C., et al. [2]	2014	Experimental Study	Viability is not essential for some beneficial effects of probiotics.
Cicenia, A., et al. [7]	2014	Clinical Study	Compounds derived from lactobacilli have beneficial postbiotic activities.
Gänzle, M.G. [10]	2014	Experimental Study	Fermentation with LAB improves the sensory and nutritional properties of bread.
De Almada, C.N., et al. [5]	2016	Review	Evidence on the ability of paraprobiotics to modify biological responses.
Salminen, S., et al. [11]	2021	Review	Definition and scope of postbiotics; health benefits and safety.

**Table 2:** Summary of regulatory opinions on attenuated microorganisms in the European Union.

EFSA NDA Panel, 2015 [27].	Neither *B. xylanisolvens DSM 23964* nor any other strain of Bacteroides has a history of use in food production and consumption. The applicant and the FSAI noted that the use of Bacteroides in food production had not been reported in the EU before May 1997 and that *B. xylanisolvens* had not been evaluated under the qualified presumption of safety (QPS) system in the moment when this novel food was evaluated by the Member States.
EFSA NDA Panel, 2021 [29].	The production process of *A. muciniphila* consists of anaerobic fermentation followed by pasteurization and concentration of bacterial cells.
EFSA NDA Panel, 2019 [28].	Inactivation of growth and death of the bacteria occurs by heating the final bacterial culture to 80 °C for 32 min. The novel food has no history of consumption in the European Union (EU).
European Commission, 2021 [32].	Human consumption of *P. freudenreichii* biomass is established to a significant extent within the Union before 15 May 1997. In the opinion of the recipient Member State, no significant change has occurred affecting its nutritional value, its metabolism or its level of adverse effects leading to the conclusion that category vii (Article 3(2)(a)(vii) of Regulation (EU) 2015/2283) is not applicable.
European Commission, 2022 [33].	The EFSA Qualified Presumption of Safety (QPS) list for *Bacillus* sp. It only applies to the production process, not as a food ingredient. No consumption history has been established before 15 May 1997 for the ingredient IBalance in the European Union that falls within the scope of Regulation (EU) 2015/2283 on novel foods.
European Commission, 2021 [35].	The application refers to a non-QPS bacteria with no history of consumption in the EU.
EFSA NDA Panel, 2022 [36].	The application refers to a non-QPS bacteria with no history of consumption in the EU.

**Table 3:** Bibliographic references attesting to the use of specific LAB before 1997 in different EU countries.

Country	Lactic Acid Bacteria	Reference(s)
Finland	*L. acidophilus*, *L. plantarum*, *L. casei*	[42]
France	*L. plantarum*, *L. casei*, *L. delbrueckii* subsp. *delbrueckii*, *L. acidophilus*, *L. brevis*, *Leuc. mesenteroides* subsp. *mesenteroides*, *Leuc. mesenteroides* subsp. *dextranicum*, *P. pentosaceus*, *L. curvatus*	[43]
Germany	*L. delbrueckii*, *L. plantarum*, *L. casei*, *L. fermentum*, *L. buchneri*, *L. brevis*	[44]
	*L. acidophilus*, *L. farciminis*, *L. alimentarius*, *L. casei*, *L. plantarum*, *L. brevis*, *L. sanfranciscensis*, *L. fructivorans*, *L. fermentum*, *L. buchneri*	[45]
	*L. acidophilus*, *L. casei*, *L. plantarum*, *L. farciminis*, *L. alimentarius*, *L. brevis*, *L. buchneri*, *L. fermentum*, *L. fructivorans*, *L. sanfranciscensis*, *Pediococcus* spp.	[46]
	*L. plantarum*, *L. casei*, *L. farciminis*, *L. homo- hiochii*, *L. brevis*, *L. hilgardii (spontaneous); L. sanfranciscensis*, *L. brevis*, *L. hilgardii*, *W. viridescens*	[47,48]
Italy	*L. brevis*, *L. plantarum*	[49]
	*L. sanfranciscensis*, *L. fermentum*, *L. plantarum*, *Leuc. mesenteroides*, *Pediococcus* spp.	[50]
	*L. sanfranciscensis*, *L. plantarum*, *L. farciminis*	[51]
	*L. sakei*, *L. plantarum*, *Leuc. gelidum*, *Leuc. Mesenteroides*	[52]
Spain	*L. brevis*, *L. plantarum*	[53]
	*L. brevis*, *L. plantarum*, *L. cellobiosus*, *Leuc. Mesenteroides*	[54,55]
Sweden	*L. fermentum*, *L. delbrueckii*, *L. acidophilus*, *L. plantarum*, *L. rhamnosus*, *L. farciminis*, *L. fermentum*, *L. sanfranciscensis*, *L. brevis*, *W. viridescens*	[56]
	*Lactobacillus* sp., *P. pentosaceus*	[57]

**Table 4:** Comparison of LAB from sourdough with those described in the scientific literature before 1997 and with the latest EFSA QPS review [58].

	<1997	QPS 2022	Sourdough
*Bacillus paralicheniformis*		YES	YES
*Bacillus subtilis*		YES	YES
*Lactobacillus acidophilus*	YES		
*Lactobacillus alimentarius*	YES		
*Lactobacillus brevis*	YES	YES	YES
*Lactobacillus buchneri*	YES		
*Lactobacillus casei*	YES	YES	YES
*Lactobacillus cellobiosus*	YES		YES
*Lactobacillus curvatus*	YES	YES	YES
*Lactobacillus delbrueckii* subsp. *delbrueckii*	YES	YES	YES
*Lactobacillus farciminis*	YES		
*Lactobacillus fermentum*	YES	YES	YES
*Lactobacillus fructivorans*	YES		
*Lactobacillus hilgardii*	YES		
*Lactobacillus homo-hiochii*	YES		
*Lactobacillus paracasei*		YES	YES
*Lactobacillus plantarum*	YES	YES	YES
*Lactobacillus rhamnosus*	YES	YES	YES
*Lactobacillus sakei*	YES	YES	YES
*Lactobacillus sanfranciscensis*	YES		
*Leuconostoc gelidum*	YES		
*Leuconostoc mesenteroides* subsp. *dextranicum*	YES		
*Leuconostoc mesenteroides* subsp. *mesenteroides*	YES		
*Pediococcus pentosaceus*	YES		
*Pediococcus* spp.	YES	YES	YES
*Weissella viridescens*	YES		

Green highlighted color means the safest and used LAB over time during bread making process.
